# Management of COPD With Cardiovascular Risk in Asia: A Review by the Asian Pacific Society of Respirology COPD Assembly

**DOI:** 10.1111/resp.70103

**Published:** 2025-08-11

**Authors:** Chin Kook Rhee, Fanny Wai San Ko, Vu Van Giap, Theerasuk Kawamatawong, Jung‐Kyu Lee, Kazuto Matsunaga, Helmy Haja Mydin, Yong‐Kek Pang, Diahn‐Warng Perng, Myla Salazar‐Supe, Yoko Shibata, David Sim, Naoya Tanabe, Augustine Tee, Hao‐Chien Wang, Yen‐Wen Wu, Faisal Yunus

**Affiliations:** ^1^ Division of Pulmonary and Critical Care Medicine, Department of Internal Medicine, Seoul St. Mary's Hospital, College of Medicine The Catholic University of Korea Seoul South Korea; ^2^ Department of Medicine and Therapeutics The Chinese University of Hong Kong, Prince of Wales Hospital Shatin New Territories Hong Kong; ^3^ Department of Internal Medicine Hanoi Medical University Hanoi Vietnam; ^4^ Respiratory Center Bach Mai Hospital Hanoi Vietnam; ^5^ Division of Pulmonary and Critical Care Medicine, Department of Medicine Ramathibodi Hospital, Mahidol University Bangkok Thailand; ^6^ Division of Pulmonary and Critical Care Medicine, Department of Internal Medicine Seoul Metropolitan Government‐Seoul National University Boramae Medical Center Seoul South Korea; ^7^ Department of Respiratory Medicine and Infectious Disease, Graduate School of Medicine Yamaguchi University Yamaguchi Japan; ^8^ Department of Respiratory Medicine, Pantai Hospital Kuala Lumpur Kuala Lumpur Malaysia; ^9^ Division of Respiratory Medicine University Malaya Medical Centre Kuala Lumpur Malaysia; ^10^ Department of Chest Medicine Taipei Veterans General Hospital Taipei Taiwan; ^11^ School of Medicine National Yang Ming Chiao Tung University Taipei Taiwan; ^12^ Department of Cardiology Philippine Heart Center Quezon City Philippines; ^13^ Department of Pulmonary Medicine Fukushima Medical University Fukushima Japan; ^14^ Department of Cardiology National Heart Centre Singapore Singapore Singapore; ^15^ Department of Respiratory Medicine Kyoto University Graduate School of Medicine Kyoto Japan; ^16^ Department of Respiratory and Critical Care Medicine Changi General Hospital Singapore Singapore; ^17^ Department of Medicine National Taiwan University Cancer Center Taipei Taiwan; ^18^ Division of Cardiology, Cardiovascular Medical Center, and Department of Medicine Far Eastern Memorial Hospital New Taipei City Taiwan; ^19^ Graduate Institute of Medicine, Yuan Ze University Taoyuan City Taiwan; ^20^ Department of Pulmonology and Respiratory Medicine, Faculty of Medicine University of Indonesia—Persahabatan Hospital Jakarta Indonesia

**Keywords:** Asia, cardiopulmonary risk, cardiovascular disease risk, chronic obstructive pulmonary disease, COPD

## Abstract

Chronic obstructive pulmonary disease (COPD) has a high burden in Asia. These patients are also susceptible to various cardiovascular diseases (CVD). A panel of expert Asian pulmonologists explored the published literature to understand the impact of COPD and CVD on each other and to identify the cardiopulmonary risk factors in the region. The experts concluded that an elevated risk of all‐cause mortality and acute cardiovascular events persists for up to 2 years following moderate and severe COPD exacerbations, with the risk of death being highest in the first 30 days after the exacerbation. High smoking rate (especially in males), high indoor and outdoor air pollution in Asia, relatively low vaccination rate in Asia (especially in low‐ and middle‐income countries), and relatively low rate of utilisation of inhaler medications impact the cardiopulmonary risk in Asia.

## 1 Introduction

Chronic obstructive pulmonary disease (COPD) is associated with significant morbidity, mortality, and socioeconomic burden, despite being treatable. The prevalence of COPD in Asia ranges between 4% and 17% [1]. According to estimates and statistical modelling, East Asia and the Pacific, as well as South Asia, are projected to be the second and third leading regions, with 124 million and 109 million COPD cases, respectively, by 2050 [2].

In COPD patients, cardiovascular diseases (CVD) are the most common comorbidity and are associated with an increased risk of mortality. The prevalence of CVD in COPD patients is estimated to be significantly higher compared with patients without COPD [3, 4]. COPD and CVD share multiple common risk factors and characteristics, such as smoking, lung hyperinflation, hypoxaemia, endothelial damage, systemic inflammation and oxidative stress, acute exacerbations, and genetics. However, the underlying mechanism leading to the co‐presentation of the two conditions is unclear [5, 6]. Furthermore, COPD and CVD may have overlapping signs and symptoms such as exertional dyspnoea, leg oedema, pulmonary oedema and pulmonary hypertension, among others [7, 8].

The data from Asia is sporadic about the causes, effects and outcomes of concomitant CVD and COPD. Further, the features of COPD patients in Asia may differ from those of the Western population, necessitating region‐specific recommendations. To address this, a panel of Asian experts reviewed existing literature and proposed recommendations for the management of concomitant CVD and COPD in Asia.

## 2 Methods

### 2.1 Literature Search and Review

A comprehensive literature search was conducted on the PubMed databases to identify relevant studies based on the objectives of the review. The review attempts to:

- Review the recent evidence in Asia on the following topics:
  - COPD hospitalisation and mortality burden in Asia.
  - The impact of COPD exacerbation beyond the lung.
  - The effect of cardiopulmonary events following an episode of COPD exacerbation.
  - Other compelling issues that are unique to the Asian region.
- Key implications due to CVD for clinical practice of COPD in Asia.
- Provide recommendations to proactively prevent COPD exacerbations and reduce mortality in Asia.

The search terms used included COPD‐related terms, cardiovascular (CV) disease‐related terms, Asia Pacific, Southeast Asia, Asia and specific Asian countries (Bangladesh, China, India, Indonesia, Japan, Malaysia, Pakistan, Singapore, South Korea, Taiwan, Thailand, and Vietnam).

The search was restricted to studies conducted in human populations and the English language; however, no time period restriction was applied. Detailed search terms and the iterations used are summarised in the Supporting Information.

### 2.2 Participants

The key members of the COPD Assembly (*n* = 14), including the leadership troika (Head, Head‐Elect, and Deputy Head) and cardiologists (*n* = 3) from Hong Kong, Indonesia, Japan, Malaysia, Philippines, Singapore, South Korea, Taiwan, Thailand, and Vietnam collaborated over a series of virtual monthly meetings between April and October 2024 to define the topics for the project, identify and review existing literature, develop recommendations and write, review, and approve the manuscript.

## 3 Results and Discussion

**FIGURE 1:**
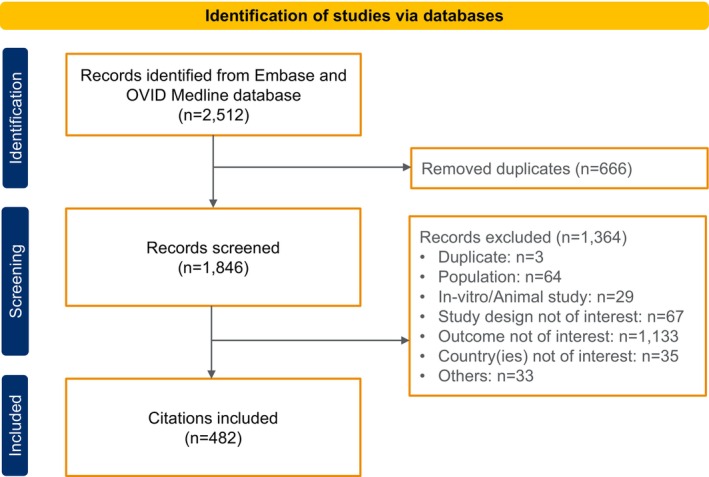
PRISMA flowchart to summarise the selection process of the eligible studies.

An overview of the results of the literature search is summarised in Figure 1.

**FIGURE 2:**
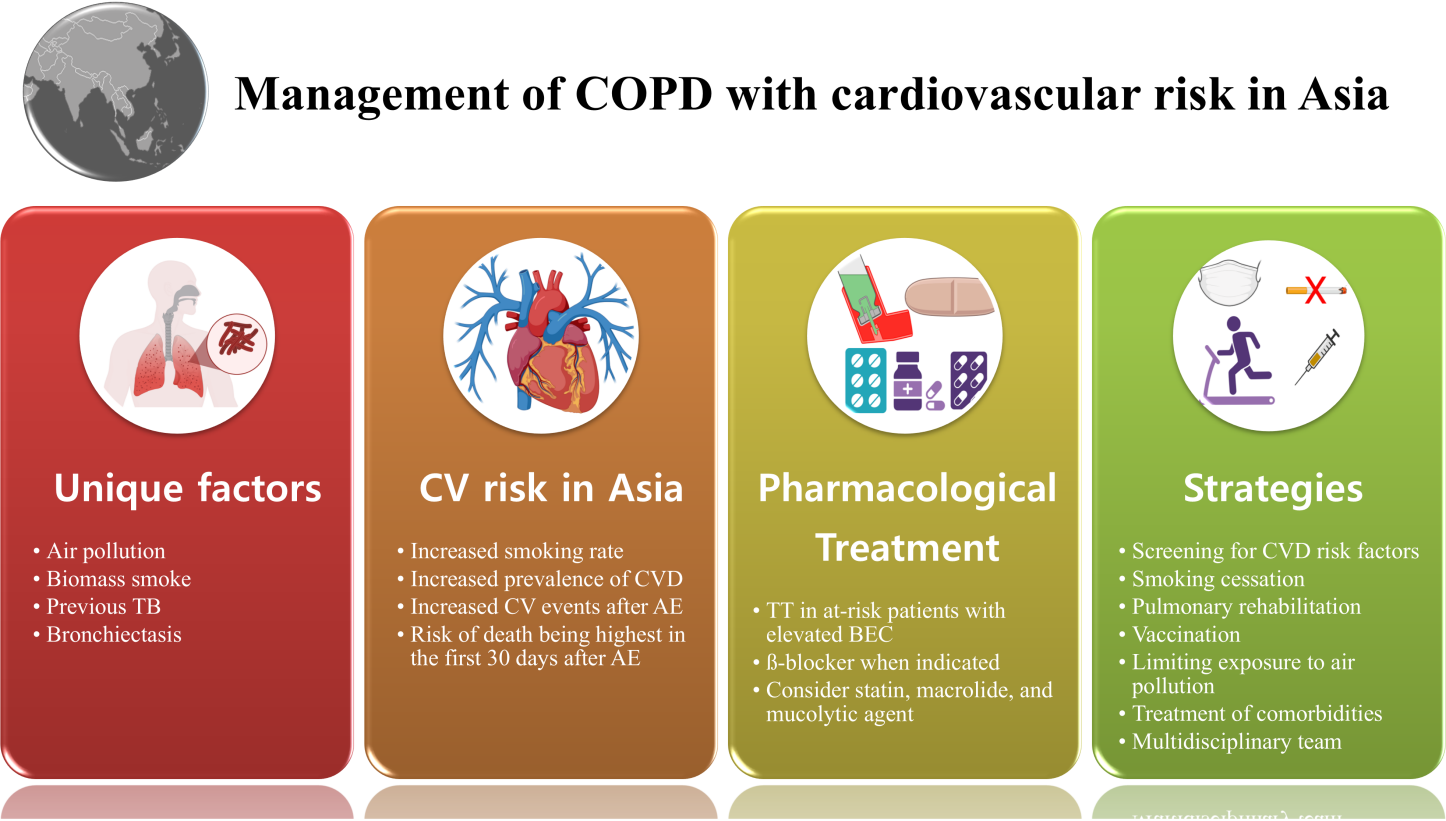
Management of COPD with cardiovascular risk in Asia. AE, acute exacerbation of COPD; BEC, blood eosinophil count; CV, cardiovascular; CVD, cardiovascular disease; DM, diabetes mellitus; HTN, hypertension; TB, tuberculosis; TT, triple therapy. This figure was created with Biorender.com.

Figure 2 summarises the Asia‐specific risk factors, cardiovascular (CV) risk, and treatment and management strategies identified and proposed by the panel members.

### 3.1 COPD in Asia

Asian patients with COPD exhibit unique clinical features compared to their counterparts in Western countries, with differences in aetiology of the disease across these regions. Factors like air pollution, biomass smoke, and indoor smoke pollution play a significant role as distinct etiological contributors in Asian countries [9, 10]. Further, poverty and various infections such as pulmonary tuberculosis, Human Immunodeficiency Virus (HIV), etc., also contribute to the pathogenesis of COPD in Asia [11]. In addition to other known risk factors, occupational exposure to inorganic dust, fumes, and particulate matter also contributes to COPD risk in Asia, especially among people working in agriculture, construction, mining, and industries [12, 13]. Additionally, climate change exacerbates the burden of COPD due to heightened exposure to air pollutants, extreme heat, and climate variability. Increased particulate matter from wildfires and urban emissions contributes to exacerbation, while heat and humidity worsen respiratory distress and increase hospitalisation. Furthermore, airway inflammation may worsen due to extended pollen seasons and mould proliferation resulting from climate shifts [14, 15, 16].

In addition to the difference in etiologies, underdiagnosis of COPD has been reported in Asia. In a study conducted in Japan, the rate of physician‐diagnosed COPD was very low among individuals who underwent health checkups (only 8.4% among subjects with airflow obstructions in spirometry had a physician diagnosis of COPD), indicating the need for increased awareness about this medical condition [17]. The proportion of under‐diagnosed COPD is likely to be high in low‐middle‐income countries for reasons such as lack of knowledge about risk factors, limited access to healthcare, asymptomatic younger individuals, under‐reporting of symptoms, and under‐reporting to a physician [18].

Underdiagnosed and undertreated COPD may result in an altered disease course. In a study conducted by RazaUllah et al. COPD was found to be underdiagnosed in patients with a high risk of coronary artery disease [19] Yang et al. reported a high burden of severe exacerbations and symptoms in Chinese outpatients with COPD, with low adherence to treatment guidelines [20] A study conducted in Bangladesh demonstrated that the prevalence of COPD was higher among males than females and in rural than urban residents. This study also demonstrated that COPD was often diagnosed at GOLD stage II, which may be indicative of a delay in diagnosis [21].

These data suggest that vigorous screening for COPD may help detect COPD at an earlier stage, which may lead to improved outcomes due to earlier intervention.

- Asian COPD patients demonstrate distinct etiologic factors as compared with non‐Asian patients.
- COPD in Asia is likely underdiagnosed, indicating a higher disease burden compared to the available data.

### 3.2 Association of COPD and Cardiovascular Disorders

CVD and COPD are both majorly age‐related and are frequently present in the elderly population. CVD encompasses a plethora of heart, blood, and vascular disorders, including coronary heart disease (CHD), congestive heart failure (CHF), cardiac arrhythmia/atrial fibrillation (AF), ischemic strokes, and venous thromboembolism (VTE), among others. The pathophysiological links between COPD and CVD are believed to be related to underlying systemic inflammation, hyperinflation, arterial stiffness, and other common risk factors. COPD is often associated with an increased risk of CV events independent of age, sex, and smoking history [4, 22, 23, 24]. Additionally, common etiologic factors for COPD, such as climate change, heat exposure, air pollution, biomass smoke, poverty, and infections, are also associated with increased prevalence of CVD [25, 26, 27]. In the Korean National Health and Nutrition Examination Survey (NHANES) 2013–2018 study, Chen et al. demonstrated that COPD patients had a significantly higher prevalence of CVD than individuals without COPD (59.6% vs. 28.4%) [24]. In an observational study conducted in India, approximately 60% of the patients with COPD had CV comorbidities, which was significantly higher compared with the non‐COPD population. The most common comorbidities included ischemic heart disease (IHD) (21%), followed by CHF (20%), stroke (5%), and arrhythmias (3%) [28]. These Asian data are consistent with studies conducted in non‐Asian populations, which demonstrated that patients with COPD were more prone to comorbidities compared with those without COPD and that the prevalence of CVD in COPD patients ranged between 25% and 70% [4, 29].

On the other hand, CVD patients are also predisposed to developing COPD. In a meta‐analysis conducted by Meng et al., the pooled prevalence of COPD was reported to be 12.0% in patients with IHD [30]. In the Chinese Heart Failure study, 11.6% of HF patients were reported to have COPD [31].

CVD in COPD patients can occur as a comorbidity, contributing to an increased frequency and severity of COPD exacerbations. Kim et al., in a study conducted in Korea, demonstrated that CHF was a major risk factor for developing exacerbation of COPD, along with other risk factors [32].

In addition to the association with stable COPD, studies have shown that exacerbation of COPD leads to a period of heightened CV risk. A large multi‐country analysis (EXACOS‐CV) demonstrated that patients with COPD experienced an elevated and sustained risk of a severe CV event or death following a moderate or severe exacerbation [33]. Further, the Japanese cohort subgroup analysis from this study demonstrated that following an exacerbation of COPD, the risk of a severe CV event was increased in the first 30 days [adjusted HR (aHR) 1.44, 95% confidence interval (CI) 1.33–1.55] and remained elevated for 365 days post‐exacerbation (aHR 1.13, 95% CI 1.04–1.23) [23]. Kunisaki et al., in the post hoc analysis from the SUMMIT trial, demonstrated that in patients with COPD who have CVD or risk factors for CVD, the occurrence of exacerbations adds an increased risk of subsequent CVD events, especially in hospitalised patients and within the first 30 days after exacerbation [34].

The presence of CV comorbidities is a predictor of the likelihood of severe exacerbations of COPD occurring in the future [32, 35]. The high prevalence of CV comorbidities in the study conducted by Athanazio et al. demonstrates that the risk of experiencing an acute CV event is significantly increased following severe exacerbations compared with non‐exacerbation periods [36]. In a study conducted in Thailand, 9% of all exacerbations were directly related to CV comorbidities [CHF (8.2%), unstable angina (0.4%), AF (0.4%)] [37].

Along with a higher prevalence of comorbidities like AF, hypertension, diabetes, hyperlipidaemia, stroke, and CHF, COPD patients are also at increased risk for other life‐threatening conditions, such as VTE. In a study conducted by Chen et al., Asian patients with COPD were found to have a higher incidence of deep vein thrombosis (DVT) than non‐COPD patients [38]. In another study by Dong et al., the risk of VTE in COPD patients increased with the degree of airway obstruction [39].

Furthermore, COPD has been shown to be a predictor of CVD mortality. Additionally, there is a correlation between lower body mass index (BMI) and higher mortality in patients with COPD [40]. In an analysis of the Asia‐Pacific Heart Rhythm Society (APHRS) AF Registry, patients with COPD had a higher incidence of all‐cause death (14.9% vs. 2.6%, *p* < 0.001), CV death (2.0% vs. 0.6%, *p* < 0.001), and heart failure (8.3% vs. 6.0%, *p* < 0.001) as compared with the non‐COPD population [41]. Shibata et al. in a study conducted in the Japanese population, demonstrated that both all‐cause and CV mortality (myocardial infarction (RR 6.70, 95% CI 2.51–17.9)) significantly increased with a worsening severity of airflow obstruction [42].

Certain variations exist in cardiopulmonary diseases among different Asian ethnicities. The Indian population, for example, has a higher prevalence of type 2 diabetes mellitus, and the Malaysian population has a higher prevalence of hypertension. These form independent risk factors for elevated cardiopulmonary risk. However, further research and evidence are desirable to conclude how the overall risk in the Asian population compares against that in the Western population.

Finally, due to the inadequate management of COPD in the Asian population, moderate or severe exacerbations often occur. Asian patients with COPD are more likely to be men, of an older age, with a lower mean BMI (< 25 kg/m^2^), and with more severe airflow limitation than those from other regions. Risk factors for COPD in Asian patients include smoking exposure of 20 pack‐years or more, exposure to annual mean particulate matter with a diameter less than 2.5 μm of 50–74 μg/m^3^ or 75 μg/m^3^ or higher, underweight, childhood chronic cough or frequent cough, and parental history of respiratory diseases. A lower risk of COPD is associated with middle or high school education and college or higher education [43, 44, 45].

In a study conducted by Tine et al., HF was reported in patients with all GOLD stages of COPD who presented to the emergency department for COPD exacerbation and was undiagnosed in approximately half of all patients [46]. These findings underline the need for routine screening for CVD in patients hospitalised for COPD‐related complications.

- Multiple CVDs are commonly associated with COPD and should be considered as significant comorbidities.
- The most associated CVD with COPD includes hypertension, dyslipidaemia, CHF, IHD, VTE, and A.
- Increased CV events after exacerbations have been observed in Asian COPD patients.
- An elevated risk of all‐cause mortality and severe CV events persists for up to 2 years following moderate and severe COPD exacerbations, with the risk of all‐cause death being highest in the first 30 days after the exacerbation.
- Even in the Asian COPD population, regional and ethnic differences exist for cardiopulmonary risk.

### 3.3 COPD Mortality Burden and Risk Factors for Mortality

**FIGURE 3:**
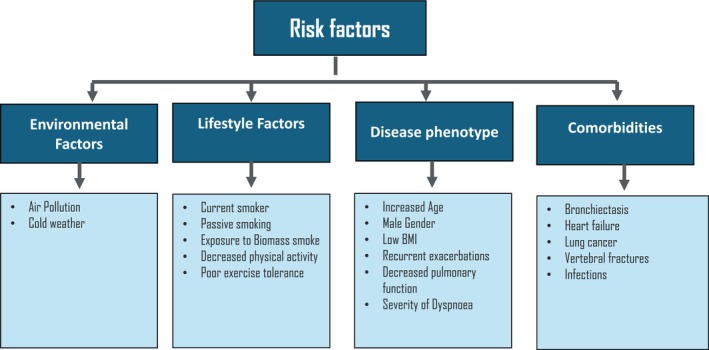
Risk factors for increased mortality in patients with COPD. BMI, body mass index.

Various risk factors have been identified which contribute to increased COPD mortality such as air pollution including fine particulate matter [47, 48, 49], exposure to cold spells [50], passive smoking [51, 52, 53], increased age [54, 55, 56], lower BMI [40, 57], recurrent exacerbations [20, 43, 58], readmission post exacerbation [58, 59], decreased pulmonary function and the subsequent complications [43], presence of bronchiectasis [54] and vertebral fractures [60] (Figure 3).

In a Korean study conducted by Kim et al. advancing age of COPD patients, male gender, low BMI and smokers with COPD had elevated risk of developing IHD. This study demonstrated a significantly higher risk of IHD in smokers with COPD compared with never‐smokers without COPD. Furthermore, COPD patients in this study had higher risk factors for developing IHD, regardless of the other common shared risk factors [61].

Furthermore, heart rate variability (HRV) with sympathetic overactivity analysis of COPD, presence of epicardial adipose tissue (EAT), elevated baseline high sensitivity CRP (hs‐CRP), and higher serum HE4 levels (Human Epididymis Protein 4) are a few of the predictive risk factors that may point towards CV mortality in COPD patients [62, 63, 64, 65].

Various biomarkers such as brain natriuretic peptide, high‐sensitivity troponins (hs‐Tn), growth differentiation factor‐15 (GDF‐15), interleukins (ILs) (IL‐6 and IL‐8), c‐reactive protein (hs‐CRP), and so on, may predict the CVD risk in COPD patients [66, 67, 68]. Further, systemic inflammation in patients with moderate to severe airflow limitation is associated with a greater risk of cardiac injury [69].

- COPD and CVD share multiple risk factors. Further, the presence of COPD itself is a risk factor for the development of CVD.
- Various biomarkers and predictive analytic tools are available for determining the risk of CVD in COPD patients. However, the evidence surrounding the use of these tools is evolving.
- The relationship between COPD medications and CV risk is complex. Patients with COPD who are at high CV risk are often prescribed medications for their COPD. While pharmacological treatment for COPD offers many benefits, it is essential for clinicians to deliver personalised therapy tailored to individual needs.

### 3.4 Socioeconomic Status in Asia and Its Impact on COPD

Socioeconomic status (SES) significantly influences health outcomes across various chronic diseases. SES encompasses more than income levels, serves as a proxy for a social or economic position or rank in a social group, and includes education, occupation, housing, assets, and participation in social organisation. Typically, lower SES is associated with higher COPD mortality and morbidity [70]. In an analysis of 3 studies in low‐middle income countries, lower education, lower household income, and lower composite SES index were associated with COPD [70]. In another study, consistent significant inverse associations between socioeconomic status and COPD outcomes were reported. Individuals of the lowest socioeconomic strata were approximately twice as likely to have poor outcomes as those of the highest [71].

A Chinese study demonstrated that white‐collar workers were less likely to experience COPD than blue‐collar workers. The study also demonstrated that education, income, and occupation had direct or indirect effects on pulmonary function mediated by smoking status, indoor air pollution, lower BMI, and outdoor air pollution [72].

In addition to SES impacting COPD outcomes, the economic burden of COPD also impacts healthcare systems. In a study conducted in Japan, annual costs of health care resource use per patient in the COPD patients in < 65‐year and ≥ 65‐year age groups were US$4389 and US$4678, respectively. Costs due to productivity loss were estimated to be US$52,870 in the < 65‐year age group and US$30,187 in the ≥ 65‐year age group. COPD represents a significant socioeconomic burden in Japan [73]. In China, patients with more severe COPD tended to use multiple bronchodilators or combine them with ICS, resulting in higher healthcare costs [74]. In another study conducted in China, an increasing trend in hospital readmission related to COPD was noted. With increasing readmission, the healthcare resource utilisation of the patients increases, thus increasing the impact on the health system [59].

Currently, guidelines and management algorithms do not factor in the effect of SES on the disease process. A standardised method needs to be created to include SES in the prognostic calculations and management of chronic pulmonary diseases.

Strategies and guidelines for mitigating these factors at individual and government levels can help to reduce the risk.

### 3.5 Pharmacological Therapy and Management

The treatment approach for patients with both CVD and COPD should be comprehensive, addressing multiple factors. It must be designed to manage both the underlying COPD and the cardiovascular disease effectively. In addition to non‐pharmacological therapies such as smoking cessation, the use of anti‐inflammatories and bronchodilators has been recommended for the treatment of COPD [75]. The use of triple therapy (TT), comprising inhaled corticosteroid (ICS)/long‐acting β2‐agonists (LABAs)/long‐acting antimuscarinic antagonists (LAMAs), has demonstrated lower mortality (including all‐cause mortality) than LAMA/LABA in COPD patients with frequent exacerbations [76, 77, 78]. In the ETHOS study, model‐estimated deaths were estimated to be 1.3% of the patients receiving TT, compared to 2.3% of patient deaths in the dual bronchodilator arm. In this study, 11 and 28 CVD‐related deaths were reported in the TT and LAMA/LABA arms, respectively. The ETHOS trial included patients from China, Japan, South Korea, and Taiwan [76, 79]. Similarly, the IMPACT study reported all‐cause deaths in 1.2% of patients in the TT arm, while 1.88% of deaths were reported in the LAMA/LABA arm. The study further reported 16 adjudicated CV‐related deaths in the TT arm, as compared to 15 in the LAMA/LABA arm (4.2 and 8.7 deaths/1000 patients). The study included patients from Hong Kong, the Philippines, Thailand, Vietnam, China, Japan, and Korea, along with other countries. This decrease in mortality is partly explained by the reduction in the number and severity of exacerbations and inflammatory events that are associated with endothelial damage [78, 80]. The subgroup analysis of Asian patients from the IMPACT trial demonstrated that ICS/LABA/LAMA‐based triple therapy was equally effective in both Asian and non‐Asian populations [78]. A study conducted in India demonstrated that TT was more effective in improving lung function and reducing mortality in COPD patients as compared to dual bronchodilator therapy [81].

Bogart et al. reported that early initiation of TT after a COPD‐related hospitalisation may reduce subsequent exacerbations and future costs [82]. Mannino et al. also reported a reduction in the rate of exacerbations in the patients who received prompt TT after an exacerbation [83]. In a study conducted in Japan, earlier initiation of TT was associated with several benefits, including decreasing the rate and prolonging the time to first or subsequent moderate‐to‐severe exacerbation, lower 90‐day all‐cause readmissions, and overall reduced healthcare resource utilisation [84].

TT can be administered either as multiple inhaler triple therapy (MITT) or as a single inhaler triple therapy (SITT). SITT is a preferred method for administration to reduce medication errors. Patients with COPD, including patients from Asia, when treated with SITT, had improved treatment persistence and adherence, as well as a risk reduction of moderate‐to‐severe exacerbation, severe exacerbation, and all‐cause mortality compared to patients treated with MITT [85, 86, 87].

Two large clinical trials for two different SITTs demonstrated a reduction in all‐cause mortality with the use of SITT [88, 89]. Wells et al. demonstrated that SITT significantly reduced the risk of composite cardiopulmonary adverse events after acute exacerbations [90]. Studies have demonstrated that the effects of TT in COPD patients are similar in Asian and non‐Asian populations [78].

Specific treatments such as β‐blockers can be utilised for controlling CVD in COPD patients [41, 91, 92, 93, 94, 95]. As demonstrated by Kubota et al., the use of β‐blockers in HF patients with COPD significantly reduced all‐cause mortality [96]. In another nationwide population‐based study by Chung et al., COPD patients prescribed cardioselective β‐blockers had lower risks of all‐cause mortality, major adverse cardiac and cerebrovascular events, and heart failure hospitalisation than COPD patients prescribed nonselective β‐blockers after MI [97]. Zhang et al. demonstrated that early initiation of β‐blockers is associated with an improvement in in‐hospital outcomes in patients with COPD after acute coronary artery syndrome [93]. β‐blockers also decrease the risks of re‐hospitalisation for COPD and other respiratory diseases by 12%–32%. The use of β‐blockers after AMI was associated with a reduced mortality risk in patients with COPD in a study conducted by Su et al. [98] Further, Wang et al. demonstrated that the use of β‐blockers is associated with a reduced risk of mortality, particularly in males, patients aged 65 years and above, and individuals with an array of comorbidities [94]. However, caution must be exercised while prescribing β‐blockers in COPD patients without a clear indication of CVD, considering that the BLOCK COPD trial demonstrated an increased risk of hospitalisation due to exacerbations in patients treated with β‐blockers [99]. Further, medications that elevate certain risk factors, such as corticosteroids, should be discontinued or adjusted to a lower dose. Where applicable, selective medications should be utilised to limit off‐target effects [96, 97, 100, 101].

Other CVD drugs, such as angiotensin II receptor blockers (ARBs) and angiotensin‐converting enzyme (ACE) inhibitors, are associated with a lower risk of exacerbations, pneumonia, and mortality in COPD patients, making them a potentially preferable choice when treatment is indicated. Additionally, statins may help reduce the risk of pulmonary hypertension in COPD, with higher daily doses providing longer‐term benefits [102, 103]. Prior use of statins and ACE inhibitors is linked to reduced mortality in patients hospitalised due to a COPD exacerbation [104]. As the severity of COPD escalates, the rise in both right ventricular global dysfunction and pulmonary hypertension increases concurrently among affected patients [68, 105, 106].

Table 1 demonstrates the effects of early initiation of various medications in patients with CVD and COPD.

- In COPD patients with frequent exacerbations, triple therapy (TT; ICS/LAMA/LABA) has been found to reduce COPD mortality more effectively than LAMA/LABA therapy and may lower the risk of cardiovascular morbidity and mortality.
- SITT is a preferred method for the administration of TT when compared to MITT, as this decreases medication errors.
- In COPD patients with cardiovascular diseases, appropriate pharmacotherapy such as β‐blockers, angiotensin II receptor blockers, and statins can help to decrease CVS risks.

**TABLE 1:** Benefits of pharmacotherapies in patients with CVD and COPD.

Therapy	Benefit
ICS/LABA/LAMA	Rates of moderate‐to‐severe and severe exacerbations following a first exacerbation were numerically lower among prompt initiators of triple therapy compared with delayed initiators of triple therapy [84].
β‐blockers	In patients with AF and COPD, the use of beta‐β‐blockers was associated with lower mortality. The association between COPD and mortality was found to be significantly modified by the use of β‐blockers [41]. β‐blockers (particularly, β‐1 selective ones) are safe and associated with reduced mortality in patients with COPD and cardiovascular disease [91]. β‐blockers‐blocker use in patients with mild COPD and AF was associated with a lower exacerbation risk [92]. The use of β‐blockers after AMI was associated with a reduced mortality risk in patients with COPD. β‐blockers did not increase the risk of COPD exacerbations [98]. The use of β‐blockers was associated with a reduced risk of mortality in most stratified analyses, particularly in males, patients aged 65 years and above, and in individuals with an array of comorbidities. These findings suggest that β‐blockers improve overall survival among COPD patients after first AMI [94]. In patients with concurrent CHF and COPD, β‐blockers reduced mortality, CHF exacerbation, and the need for hospitalisation. Bisoprolol reduced mortality and CHF exacerbation compared to carvedilol and metoprolol [95].
Statins	The use of statins was associated with significantly lower risks of acute exacerbation and also in patients with bronchiectasis‐COPD overlap [103]. Post‐AMI including statins reduced mortality risk [100].
Macrolides	The use of macrolides was associated with significantly lower risks of acute exacerbation and also in patients with bronchiectasis‐COPD overlap [103].
Mucolytic	The use of mucolytic agents was associated with significantly lower risks of acute exacerbation and also in patients with bronchiectasis‐COPD overlap [103].
Antiplatelets	Post‐AMI including antiplatelets reduced mortality risk [100].
ACEI/ARB	Post‐AMI including ACEI/ARB reduced mortality risk [100].
Xanthiums	In patients with acute AMI, xanthiums were associated with a reduced mortality rate in AMI and NSTEMI cohorts [100].

Abbreviations: ACEI, angiotensin converting enzyme inhibitor; AF, atrial fibrillation; AMI, acute myocardial infarction; ARB, angiotensin II receptor blocker; CHF, congestive heart failure; COPD, chronic obstructive pulmonary disorder; LABA, long‐acting β2 agonists; LAMA, long‐acting muscarinic antagonist; NSTEMI, non‐ST‐elevation myocardial infarction; SABA, short acting beta agonists; SAMA, short‐acting muscarinic antagonists; STEMI, ST‐elevation myocardial infarction.

### 3.6 COPD Discharge Care Bundle

A COPD discharge bundle (also called a disease management program, discharge protocol, care bundle, etc.) is a set of evidence‐based practices aimed at improving patient outcomes after discharge from acute exacerbation of COPD. Appropriate inpatient care, proper discharge management, and post‐discharge support are crucial factors to reduce early readmission in patients with COPD. Some interventions, including early follow‐up care and a care bundle after hospital discharge, can effectively reduce these early readmissions. In a study conducted by George et al., patients were provided with disease management programmes (DMPs), which included education about the disease, optimisation of evidence‐based medications, information and support from case managers, and the institution of self‐management principles. Participation in the DMP was associated with lower all‐cause mortality when compared to the controls. However, this survival gain in the programme patients was paradoxically associated with an increase in readmission rate and total hospital days [107].

One of the recommendations for inclusion in the discharge bundle is retraining patients' inhaler technique, and patient education regarding the role of the maintenance regimen and completion of steroid therapy and antibiotics, if prescribed. The necessity for long‐term oxygen therapy should be re‐evaluated. A follow‐up visit at 4–8 weeks after discharge should be arranged, and a plan for comorbidity management and follow‐up should be provided [108]. Another recommendation for a care bundle states the inclusion of continuity of care with a primary care physician or pulmonologist, optimisation of medication, supervision of inhaler use, assessment and management of comorbidities, smoking cessation, and referral to pulmonary rehabilitation [59]. However, important care gaps in the Asia Pacific remain with regard to discharge care bundles for patients with COPD; possible causes include resource constraints and lack of proper reimbursement models [109].

For patients with suspected CV diseases, appropriate non‐invasive investigations, such as echocardiography, stress tests such as treadmill test, stress echocardiography, stress myocardial perfusion imaging, coronary imaging including coronary calcium score (CCS) or coronary computed tomography angiography (CCTA), measurement of N‐terminal pro‐brain natriuretic peptide (NTproBNP) and Holter examination, can be considered for early diagnosis of CVD and further management [68, 110, 111]. Additionally, patients with high CV risk or pre‐existing CVD can be referred to a cardiologist. Combined care by a primary care physician, a respiratory physician and a cardiologist could be considered.

- A discharge care bundle can be categorised into: Therapies (COPD & non‐COPD), Testing (COPD & CVD) and Continuity of Care (follow‐up visits, scheduled appointments, education, etc.), enabled by coordinated access to healthcare information from providers.
- Routine chest x‐rays and electrocardiograms are recommended, while echocardiography and NTproBNP (or BNP) can be considered in case of progressive dyspnoea.
- In case of exertional angina or findings of coronary calcification on computed tomography (CT), stress test or CCTA can be considered.
- In case of frequent palpitation, 24‐h Holter or longer duration (e.g., 7 or 14‐day) continuous ECG recording can be considered for the detection of cardiac arrhythmias, especially AF.

### 3.7 Healthcare Optimisation and Multidisciplinary Management

Patients who were recently hospitalised for COPD exacerbations should be followed up at regular, short intervals. Post‐discharge pulmonary rehabilitation and comprehensive care, including optimisation of the management of comorbidities, can help to decrease future exacerbations [112]. According to the GOLD 2024 recommendations, FEV_1_ by spirometry should be assessed at least once a year to identify patients with lung function decline [113]. In patients with concomitant COPD and CVD, heart rate variability (HRV) analysis may be important for planning novel therapeutic and preventive approaches in the care of patients with COPD [64]. Measurement of aortic pulse wave velocity (aPWV) to assess arterial stiffness may be a useful tool, as frequent COPD exacerbators tend to have greater arterial stiffness than infrequent exacerbators [114]. However, more studies would be needed to assess their applications in the management of COPD patients.

A collaborative approach between cardiologists and pulmonologists may be beneficial to achieve optimal management of patients with CVD and COPD.

- A multi‐disciplinary approach should not only emphasise pharmacological treatments like triple therapy but also include vaccination and pulmonary rehabilitation. Collaboration with other healthcare providers and seamless transitions within the care delivery route are necessary for providing effective care. Communication among healthcare providers is crucial for improving understanding of each other's roles and enhancing mutual support.
- Healthcare providers should recognise the importance of assessing and managing cardiovascular risk in COPD patients, raise awareness, and address CV risk to reduce mortality and the overall healthcare burden. Routine screening for CVD risk factors should be systematically implemented as a preventative measure to reduce cardiopulmonary risk in Asian COPD patients.
- For patients with CVDs, spirometry, in addition to cardiovascular investigations, would be important for assessment and monitoring lung function

### 3.8 Strategies and Guidelines

The current approach to managing COPD is often reactive rather than proactive. Additionally, treatments are frequently not adjusted when treatment goals are not met, or ineffective treatments are continued. The inadequate management of COPD is compounded by late diagnosis and insufficient awareness of cardiopulmonary risk [115].

The definition and terminology of ‘cardiopulmonary risk’ vary across the literature, which demonstrates inconsistencies in usage and understanding of this term. Singh et al. defined cardiopulmonary risk as ‘the risk of serious respiratory and/or CV events in patients with COPD’ and included COPD exacerbations, myocardial infarction, stroke, heart failure decompensation, arrhythmia and death due to any of these events under the lexicon [115]. The literature surrounding cardiopulmonary risk is limited and requires further deliberation and thought. Guidelines for managing COPD should draw a wider perspective to address CV disease and the prevention of CV events, considering factors such as population ageing and the impact of air pollution. The European Society of Cardiology guidelines on CVD prevention recommend estimating the 10‐year CV risk in these patients using SCORE2 tables [116]. This approach is likely also suitable for patients with comorbid COPD. A recent study conducted by Amegadzie et al. demonstrated that the COPD CVD risk stratification tools such as QRISK3 are susceptible to underestimating the risk of CV events in COPD patients [117]. In 2024, a new tool entitle QR4 was introduced by Hippisley‐Cox et al. which demonstrated improved CVD risk prediction as well as identifying new risk factors as compared to older tools, including QRISK3 and SCORE2 [118]. These tools require further validation in the context of COPD to improve risk stratification and prediction of total cardiovascular risk in patients with COPD.

Table 2 summarises various strategies suggested for reducing exacerbation and mortality burden in patients with CVD and COPD, including smoking cessation [119], indoor and outdoor pollution control [120, 121, 122], vaccination [123, 124, 125], lifestyle modification [126] and tackling adverse effects of socioeconomic status [127].

**TABLE 2:** Strategies to implement to reduce exacerbations and mortality burden.

Category	Strategy
Smoking cessation, pulmonary rehabilitation and self‐management programs	Smoking cessation, pulmonary rehabilitation, and self‐management interventional programs are important strategies for the prevention of COPD exacerbations [119].
Indoor and outdoor air pollution	Clean air can potentially decrease COPD morbidity and mortality. This protective effect is primarily attributed to the reduction in particulate matter concentration [120]. Reducing PM2.5 concentrations might be a viable approach to reduce COPD exacerbation admissions and associated healthcare burden [121]. To improve air quality, governments should enforce stricter regulations on the use of coal and biofuels, while also encouraging the use of clean fuels. Homeowners should be incentivised to instal ventilation equipment and upgrade their stoves to reduce the emission of incomplete combustion byproducts. This is particularly crucial for residents living in rural areas [9]. More benefits could be achieved from the continuous reduction of air pollution. Public efforts to prevent COPD should include the reduction of ambient air pollution [122].
Vaccination of individuals with COPD	Vaccination coverage needs to be increased in patients with COPD exacerbation, as well as for patients with CVD [123]. Appropriate and timely vaccination programs are recommended for COPD patients as in the general elderly population [124]. Influenza vaccination was associated with a decreased risk of respiratory failure in patients with COPD. Recommendations for annual vaccination (influenza, pneumococcal, COVID‐19, tetanus–diphtheria–acellular pertussis, RSV, and zoster vaccines) should be made when managing this high‐risk patient group [75, 125].
Lifestyle modification	Population‐level interventions targeting reductions in physical inactivity, obesity, and malnutrition play a supportive role in improving outcomes related to disease exacerbations [126].
Socioeconomic status	Strategies to mitigate the adverse effects of poor socioeconomic factors at individual and government levels, such as better surveillance targeting diagnosed and undiagnosed COPD in disadvantaged people, education to improve awareness and minimise exposure to risk factors of COPD, and improving access to healthcare, can potentially help improve COPD patients' outcomes [127].

Abbreviations: COPD, chronic obstructive pulmonary disease; CVD, cardiovascular disease; PM2.5, particulate matter 2.5; RSV, respiratory syncytial virus.

## 4 Conclusion

Asian COPD patients demonstrate distinct risk factors and patterns of disease. The demographic characteristics, socioeconomic status, and healthcare systems in the region are also diverse and differ from those in Western countries. Considering the unique features of the population and the challenges faced by physicians in the region, a tailored approach is desirable for the optimum management of patients with concomitant COPD and CVD.

## Conflicts of Interest

C.K.R. has received consulting fees from AstraZeneca, GSK, Novartis, Sanofi; honoraria from MSD, AstraZeneca, GSK, Novartis, Takeda, Mundipharma, Boehringer Ingelheim, Teva, Sanofi, Organo, Roche, and Bayer. F.W.S.K. has received funding for clinical research from AstraZeneca, Boehringer Ingelheim, GlaxoSmithKline, and Novartis, with all fees paid directly to her institution. F.W.S.K. is an Editorial Board member of *Respirology* and was excluded from all editorial decision‐making related to this article. K.M. received lecture fees from AstraZeneca, GlaxoSmithKline, Kyorin Pharma, Novartis Pharma, and Sanofi. M.S.‐S. has received honoraria from Menarini, Abbott Philippines, AstraZeneca, Bayer Philippines, Boehringer‐Ingelheim, Corbridge, Dega Philippines, Getz Pharma, LRI‐Therapharma, Natrapharm, Novartis, Novo Nordisk, Torrent, Zydus; travel and meeting support from Menarini, Bayer, Natrapharm; serves on the board for the Philippine Society of Echocardiography, Philippine Heart Association, Philippine Lipid and Atherosclerosis Society, and owns stocks in Metronorth Hospital and Medical Center. Y.S. received lecture fees from AstraZeneca, Boehringer Ingelheim, and GlaxoSmithKline. N.T. received lecture fees from AstraZeneca, GSK, and Sanofi and research funding from Fujifilm and Daiichi Sankyo. A.T. received honoraria fees for ad hoc local COPD advisory boards under AstraZeneca, GlaxoSmithKline, and Sanofi. Received research grants to institutions from GlaxoSmithKline and AstraZeneca. F.Y. has received speaker fees and meeting travel support from Zuellig Pharma, GSK, AstraZeneca, and Wellesta Indonesia; travel support from Zambon; has participated in advisory board meetings for AstraZeneca and GSK. V.V.G., T.K., J.‐K.L., Y.‐K.P., D.S., H.‐C.W., Y.‐W.W., H.H.M. and D.‐W.P. declared no conflicts for this work. AstraZeneca provided support for virtual meetings and funding for a medical writer to assist with the literature review and writing process. AstraZeneca had no involvement in the content of the manuscript and did not participate in any part of the writing process.

## Supporting information


**Data S1:** Supporting Information.

## Data Availability

No datasets were generated during the development of this article.
