# Supplementary material for: Management of COPD With Cardiovascular Risk in Asia: A Review by the Asian Pacific Society of Respirology COPD Assembly
Source: Respirology. 2025 Aug 11;30(9):817–30. doi: 10.1111/resp.70103 (PMC12438046; doi:10.1111/resp.70103)
Supplement: Supplementary file 1 — Data S1: Supporting Information. [file RESP-30-817-s001.docx]

**Management of COPD with cardiovascular risk in Asia: A Review by the Asian Pacific Society of Respirology COPD Assembly**

**Supplementary material**

**Search terms**

| **PICOS** | **Search terms** |
| --- | --- |
| **Population** | ("Chronic Obstructive Airway Disease" or "Chronic obstructive pulmonary disease" or "Chronic obstructive pulmonary diseases" or "Chronic obstructive pulmonary diseases" or COPD or COAD or COBD or AECOPD) |
|  | ("bronchial neoplasms" or "bronchiolitis" or "cystic fibrosis" or "interstitial lung disease" or "lung neoplasms" or "bronchopulmonary dysplasia" or cancer or neoplasm or "acute bronchitis") |
| **Outcomes** | ("Position statement" or "Asian Pacific Society of Respirology" or Consensus or recommendation* or guideline* or Statement* or "expert opinion") |
|  | ((Exacerbation or exacerbations) and (moderate or severe or "moderate to severe" or "moderate-to-severe")) |
|  | (Cardiopulmonary or Cardiovascular) |
|  | ("Major adverse cardiovascular event" or "Major adverse cardiovascular events" or MACE or "Cardiopulmonary risk" or "Cardiovascular risk" or "Cardiovascular comorbidity" or "Cardiovascular event" or "Cardiovascular events" or "Cardiovascular risk factor" or "Cardiovascular risk factors" or "cardiovascular disease") |
|  | (Mortality or morbidity or death or died) |
|  | ("Pharmacological treatment" or Pharmacotherapies or Pharmacotherapy or "triple therapy") |
|  | (Smoke-induced or "Cigarette smoke" or "Smoking cessation" or Smokers or Smoking or "Particulate Matter") |
| **Publication type limit** | (book* or chapter* or editorial or erratum or letter or note or "short survey" or "case reports" or comment or news or review) |
|  | (Costs or Cost-effectiveness or "Cost effectiveness" or Cost-utility or "Cost utility" or Cost-minimization or "Cost minimization" or "Cost analysis" or "Cost impact" or "direct cost" or "direct costs" or "indirect cost" or "indirect costs" or "budget impact" or "economic burden" or "economic outcomes" or "economic outcome" or "economic impact" or "economic evaluation" or "economic analysis" or Pharmacoeconomic or "modelling study" or "cost" or "economic") |
| **Countries** | (South Korea or Japan or China or Taiwan or Vietnam or Thailand or Singapore or Malaysia or Indonesia or India or Pakistan or Bangladesh or "Hong Kong").mp. or (Asia or Asia-pacific or "Asia pacific" or APAC or "South-east Asia" or "South east Asia" or "Southeast Asia" or "South-east Asian" or "South east Asian" or "Southeast Asian" or SEA or Asian) |
